# Socioeconomic and environmental determinants of dengue transmission in an urban setting: An ecological study in Nouméa, New Caledonia

**DOI:** 10.1371/journal.pntd.0005471

**Published:** 2017-04-03

**Authors:** Raphaël M. Zellweger, Jorge Cano, Morgan Mangeas, François Taglioni, Alizé Mercier, Marc Despinoy, Christophe E. Menkès, Myrielle Dupont-Rouzeyrol, Birgit Nikolay, Magali Teurlai

**Affiliations:** 1 Faculty of Infectious and Tropical Diseases, London School of Hygiene & Tropical Medicine, London, United Kingdom; 2 Epidemiology of Infectious Diseases Expertise and Research Unit, Institut Pasteur in New Caledonia, Institut Pasteur International Network, Nouméa, New Caledonia; 3 IRD, UMR ESPACE-DEV (UR/UA/UG/UM/IRD), Nouméa, New Caledonia; 4 University of Reunion Island, UMR Prodig/OIES (Cregur), Reunion Island, France; 5 CIRAD/INRA, UMR Contrôle des Maladies Animales Exotiques et Emergentes (CMAEE), Montpellier, France; 6 IRD / Sorbonne Universités (UPMC, Université Paris 06) / CNRS / MNHN, LOCEAN – UMR 7159, Nouméa, New Caledonia; 7 Dengue and Arboviruses Expertise and Research Unit, Institut Pasteur in New Caledonia, Institut Pasteur International Network, Noumea, New Caledonia; 8 Mathematical Modelling of Infectious Diseases Unit, Institut Pasteur, Paris, France; 9 CNRS, URA3012, Paris, France; 10 Center of Bioinformatics, Biostatistics and Integrative Biology, Institut Pasteur, Paris, France; Oregon Health and Science University, UNITED STATES

## Abstract

**Background:**

Dengue is a mosquito-borne virus that causes extensive morbidity and economic loss in many tropical and subtropical regions of the world. Often present in cities, dengue virus is rapidly spreading due to urbanization, climate change and increased human movements. Dengue cases are often heterogeneously distributed throughout cities, suggesting that small-scale determinants influence dengue urban transmission. A better understanding of these determinants is crucial to efficiently target prevention measures such as vector control and education. The aim of this study was to determine which socioeconomic and environmental determinants were associated with dengue incidence in an urban setting in the Pacific.

**Methodology:**

An ecological study was performed using data summarized by neighborhood (i.e. the neighborhood is the unit of analysis) from two dengue epidemics (2008–2009 and 2012–2013) in the city of Nouméa, the capital of New Caledonia. Spatial patterns and hotspots of dengue transmission were assessed using global and local Moran’s I statistics. Multivariable negative binomial regression models were used to investigate the association between dengue incidence and various socioeconomic and environmental factors throughout the city.

**Principal findings:**

The 2008–2009 epidemic was spatially structured, with clusters of high and low incidence neighborhoods. In 2012–2013, dengue incidence rates were more homogeneous throughout the city. In all models tested, higher dengue incidence rates were consistently associated with lower socioeconomic status (higher unemployment, lower revenue or higher percentage of population born in the Pacific, which are interrelated). A higher percentage of apartments was associated with lower dengue incidence rates during both epidemics in all models but one. A link between vegetation coverage and dengue incidence rates was also detected, but the link varied depending on the model used.

**Conclusions:**

This study demonstrates a robust spatial association between dengue incidence rates and socioeconomic status across the different neighborhoods of the city of Nouméa. Our findings provide useful information to guide policy and help target dengue prevention efforts where they are needed most.

## Author summary

Dengue virus is rapidly spreading throughout tropical and subtropical regions worldwide, possibly aided by environmental change, urbanization and/or increase in human mobility. Already present in 120 countries, dengue virus causes extensive disease burden and generates large economic costs. As dengue is mosquito-borne, its transmission pattern is strongly influenced by climate. However, dengue cases are not always distributed evenly throughout cities, where climate can be assumed to be homogenous. This suggests that other factors which are heterogeneously distributed in cities could play a role in dengue transmission, such as socioeconomic status and environmental factors (both natural and built). Identifying those factors is crucial to develop and target dengue prevention interventions, such as mosquito control and education. Our study uses dengue incidence statistics from two large epidemics in Nouméa, the capital of New Caledonia, to investigate which socioeconomic or environmental factors correlate with dengue incidence in an urban setting. Dengue incidence was consistently higher in neighborhoods where socioeconomic status was lower (i.e. lower revenue or higher unemployment) and often higher where the proportion of single-family houses in all buildings was higher. Our data suggest that, if resources are limited, prevention measures should be targeted in priority towards neighborhoods of lower socioeconomic status.

## Introduction

Dengue virus is a rapidly spreading mosquito-borne virus of global public health importance [1–3], causing extensive morbidity and economic loss in tropical and subtropical regions [2, 4, 5]. Dengue is present in over 120 countries, and estimates suggest as many as 390 million infections per year worldwide, a quarter of which are symptomatic [6–8]. Possible reasons for this rapid expansion include: climate change, population growth, urbanization, increased domestic and international travel, as well as disruption of health systems and vector control [1, 9–11]. Vaccine development efforts are ongoing [12–18], but education and vector control remain crucial for dengue prevention.

The Pacific region has recently experienced increasingly frequent epidemics of dengue, chikungunya and Zika viruses, all transmitted by *Aedes* mosquitoes [19–21]. From September 2008 to August 2009, New Caledonia suffered an epidemic with 8,586 reported dengue cases [22], initially caused by dengue serotype 1 (DENV1), until dengue serotype 4 (DENV4) started to co-circulate, causing the majority of reported cases [19]. In 2012–2013, New Caledonia experienced the largest outbreak documented on its soil with 11,001 reported cases [22] mainly caused by DENV1 [19]. The only known vector of dengue virus in New Caledonia is *Aedes aegypti* [23, 24] a human-biting mosquito that lives in peri-domestic urban environments, bites indoors and outdoors, particularly at dawn and dusk, and clusters around man-made breeding sites such as open containers with stagnant water [9, 25].

Resulting from complex virus-host-vector-interactions, dengue transmission is strongly linked to climate (rainfall, humidity and temperature [9, 26–29]), natural and built environment, human movements and behaviors, as well as immune status of the population [1, 25, 30–32]. In urban settings, clustering of dengue cases ranges from small household foci to larger neighborhood clusters [25, 30, 31, 33–45]. The heterogeneous spatial distribution of cases within cities, where climate can be considered uniform, indicates that further small-scale, non-randomly distributed determinants of transmission exist.

Previous studies have suggested that dengue was associated with: (i) low socioeconomic status, low income and/or low education [33, 38, 43, 46–52], (ii) low literacy [39, 43], (iii) lack of knowledge about dengue [48, 53], (iv) presence of economically inactive people in the household (unemployed, students, house workers) [39, 47], (v) household crowding (number of people per room) [50, 53], (vi) poor housing (inadequate sewage and garbage disposal, lack of screens and air-conditioning) [33, 37, 46, 47], (vii) household density [38, 44], and (viii) type of housing (individual house versus apartments, large residential area) [43, 50, 54, 55].

However, other studies found no association between dengue and income or number of people per room [56], or concluded that dengue affected preferentially higher socioeconomic groups [35, 57]. These contradictory results, potentially due to heterogeneity in measurements of dengue burden and socioeconomic indicators [58], underscore the need for local studies to better understand setting-specific determinants of transmission. While many studies have investigated socioeconomic determinants of dengue transmission in the Americas, few have done so in the very different geographical setting of the Pacific.

The aim of this study was to identify socioeconomic and environmental factors associated with dengue incidence in an urban setting in the Pacific region. We performed an ecological study (with the neighborhood as the unit of analysis) to describe spatial associations between dengue incidence rates and various socioeconomic and geographic factors during two major dengue epidemics in Nouméa (New Caledonia). Our results will help targeting prevention efforts aimed at reducing transmission of dengue virus and other viruses transmitted by the *Aedes* mosquito within urban settings in the Pacific region.

## Methods

### Study site

New Caledonia is an archipelago situated in the subtropical southwest Pacific Ocean, between 19° and 23° of south latitude. The main island (called “La Grande Terre”) is a 400 km long and 50–70 km wide mountainous island, about 1200 km east of Brisbane (Australia). New Caledonia has a subtropical climate, with a warm and wet season from December to March (average temperature 26–27°C), and a cool and dry season from June to September (average temperatures 19–21°C) [24, 26]. The population of around 250,000 is composed of native Melanesians (40%), people of European descent (30%), the rest coming from other Pacific Islands or Asia [59]. The capital Nouméa (22°16′33″S 166°27′29″E) is a coastal city on a hilly peninsula near the southern tip of “La Grande Terre”. Nouméa covers around 50 square-km and is administratively divided in 37 official neighborhoods (S1 Fig). The population of Nouméa was 97,579 inhabitants in 2009 [60]. Population, surface area and population density are listed for all neighborhoods in S1 Table.

### Epidemiological data

Number of dengue cases in each neighborhood in Nouméa from September 1st 2008 to August 31st 2009 and from September 1st 2012 to August 31st 2013 were obtained from the New Caledonia health authorities website [22]. Dengue incidence rates (cases per 1000 person-years (P-Y)) for both epidemics were calculated based on the number of dengue cases in each neighborhood, the population reported for each neighborhood during the 2009 census [60] and an at-risk period of one year for each epidemic.

### Case definition

Reporting dengue cases to the New Caledonian health authorities is required by law. Cases are defined according to following criteria [61]: (i) possible case: dengue clinical signs and dengue-specific-IgM positive serology outside of an epidemic period, (ii) probable case: same as possible case, but during an epidemic and (iii) confirmed case: dengue clinical signs and either dengue-PCR positivity, dengue-NS1 positivity or dengue-specific-IgM-seroconversion. Clinical signs include: headache, retro-orbital pain, joint and muscle pain, fatigue, maculo-papular rash, vomiting, limited hemorrhage [61]. Case numbers reported by the New Caledonian health authorities and used in this study include possible, probable and confirmed cases.

### Geographic and socioeconomic data

The administrative boundaries of New Caledonia were obtained from the Global Administrative Areas database (http://www.gadm.org/) while the administrative boundaries of the neighborhoods of Nouméa were provided by the Direction of Infrastructure, Topography and Terrestrial Transportation (DITTT).

Socioeconomic and geographic factors included in the analysis, as well as their median, 5- and 95-percentile are presented in Table 1. They were selected according to following rationale: (i) higher vegetation coverage may provide more breeding sites for mosquitoes and increase larvae survival (shade maintains water in breeding sites); (ii) higher number of people per room (household crowding) may increase the chance of mosquitoes feeding on multiple targets, which increases the chance of transmission; household and population density were chosen for similar reasons; (iii) older and more degraded lodgings may increase opportunities for mosquito-human contacts (fewer window screens, windows and doors closing less tightly, no air conditioning); percentage of cement lodging was chosen for similar reasons; (iv) apartments were taken as a proxy for a predominantly indoors lifestyle, and an environment that offers fewer mosquito breeding sites; (v) socioeconomic indicators (e.g. unemployment, revenue, education) were selected based on review of the literature, and internet access was chosen as a proxy of material wealth; (vi) place of birth (born in the Pacific vs the many people born elsewhere who immigrated more recently to New Caledonia) was selected as it could influence lifestyle, for example the amount of time spent outdoors versus indoors or habits concerning personal protection against mosquitoes; (vii) age was selected as a possible confounder (age could be linked to socioeconomic status, and also to the likelihood of developing dengue).

**Table 1:** Variables used in regression analysis.

Variable	Description	Median^(a)^	Percentile	
			5%	95%
**Vegetation coverage**				
Vegetation coverage 2008	Percent of surface covered by vegetation in 2008^(b)^	18.2	1.2	65.2
Vegetation coverage 2013	Percent of surface covered by vegetation in 2013^(b)^	24.1	0.8	64.4
**House and people density**				
Household crowding	Average number of inhabitant per room^(c)^	0.8	0.6	1.4
Household density	Number of households per square kilometer^(c)^	830.0	58.1	2468.5
Population density	Number of inhabitants per square kilometer^(c)^	2791.5	296.3	5675.6
**Built environment**				
Old buildings	Percentage of buildings built before 1990^(d)^	57.8	10.0	97.6
Degraded lodgings	Percentage of lodgings that are degraded^(d)^	2.95	0.0	25.9
Apartments	Percentage of apartments in all lodgings^(d)^	56.3	4.0	88.6
Cement lodgings	Percentage of lodgings with cement walls^(d)^	92.7	39.6	98.1
**Socio-economic status**				
Unemployment	Unemployment index^(^^d^^,^^e^^)^	4.3	1.1	12.9
Low education	Percent people with education below high school^(d)^	56.8	30.2	87.8
Revenue	Median monthly revenue^(^^d^^,^^f^^)^	197,500	83,000	338,000
Difference in revenue	Revenue difference lowest and highest decile^(^^d^^,^^f^^)^	460,000	218,000	812,000
Internet home access	Percent of households with home internet access^(d)^	53.3	5.0	78.4
**Demographics**				
Born in the Pacific	Percent of residents born in the Pacific Islands^(d)^	66.9	22.6	97.4
Age	Average age of the neighborhood population^(d)^	33.2	26.5	43.1

^(a)^ median and percentiles for year 2009 if not specified otherwise

^(b)^ see supporting information for the calculation of vegetation coverage

^(c)^ variables calculated from census data and neighborhood surface area, as described in the text

^(d)^ variables obtained directly from census data, already summarized by neighborhood

^(e)^ percent people looking for employment on September 30th in the population aged 15 and above

^(f)^ in Franc des Colonies Françaises du Pacifique (FCFP), 1000 FCFP ≈ 8.5 € at the time of writing

Socioeconomic indicators for each of the neighborhoods in Nouméa were obtained from the 2009 New Caledonia census. The results of the census are available summarized for all neighborhoods on the New Caledonia Institute for Statistics and Economical Studies website (ISEE-NC) [60]. Percentage of surface area covered by vegetation during the two dengue epidemics was generated for each neighborhood from remote sensing images (see Supporting Information and S2 Fig). All other variables were obtained from the census, either “as is” or generated as follow: (i) household and population densities were calculated using total household and population count in each neighborhood (2009 census) divided by the surface area of each neighborhood; (ii) average number of people per room was obtained for each neighborhood by dividing the average number of people per household by the average number of rooms in each house. For variables related to the human population (e.g. age, revenue, birthplace), we used either a summary measure for the whole population of each neighborhood (e.g. average age of the whole population in each neighborhood, median revenue of the whole active population in each neighborhood) or a percentage for each neighborhood (e.g. percentage of the population born in the Pacific in each neighborhood, percentage of the population with a lower education level in each neighborhood) as available in the census.

### Spatial analysis of dengue incidence rates

Choropleth maps representing the incidence rate of dengue in each neighborhood relative to the average incidence rate throughout the entire city were generated using QGIS 2.10 (http://www.qgis.org). The presence of spatial autocorrelation of dengue incidence rates throughout the neighborhoods was assessed using the global Moran’s *I* statistic [62]. Clusters of neighborhoods of high or low incidence were identified using the local Moran’s *I* statistic, a local indicator of spatial association (LISA) [63]. Both local and global Moran’s *I* were computed using a weighing matrix based on a Euclidian distance of 4km using the software GeoDa 1.6.7 [64].

### Statistical modelling

An ecological study (with the neighborhood as unit of analysis) using univariable and multivariable generalized linear modelling was performed to investigate the spatial association between dengue incidence rates (outcome variable) and various socioeconomic and environmental factors (explanatory variables). The 2008–2009 and 2012–2013 epidemics were analyzed separately. The unpopulated neighborhood Koumourou was excluded from the analysis. Therefore, the analytical sample size for our ecological study was 36 neighborhoods (37 official neighborhoods in Noumea, minus one excluded).

We fitted models based on the negative binomial function, which is appropriate for the analysis of over-dispersed disease count data [65, 66], and has been used previously to analyze dengue incidence data [65–69]. The majority of variables were more closely associated with dengue incidence rates once categorized into quintiles rather than as continuous variables (based on likelihood ratio test). Most variables in quintiles did not show departure from linearity (based on the departure from linearity test). Therefore, to maintain consistency across variables and comparability between the two epidemics, all explanatory variables were categorized into ordered quintiles for analysis as described in S2 Table. Associations of dengue incidence with explanatory variables were reported as incidence rate ratios (IRR), which describe the relative increase or decrease in incidence rate when the explanatory variable increases from one quintile to the next. Statistical analysis was performed using R 3.2.0 [70].

### Multivariable modelling

All variables were first tested in univariable analysis. Subsequently, they were all included in the multivariable model selection procedure, regardless of their association with the outcome. However, to reduce multicollinearity in the statistical model, we first assessed pair-wise correlations between explanatory variables before proceeding to the actual backward elimination regression modelling. Within a group of correlated variables, only the most strongly associated variable (based on the likelihood ratio test) was further used for multivariable modelling. All variables related to socioeconomic status, as well as the percentage of people born in the Pacific, were strongly correlated (S3 Table). From this group of variables, only unemployment (for 2008–2009) and revenue (for 2012–2013) were included for further multivariable modelling. Similarly, household density and population density were highly correlated, and only household density was used for multivariable modelling for both epidemics. All remaining variables had pairwise correlation coefficients between -0.7 and 0.7, and were therefore included for further multivariable modelling, regardless of their degree of association with the outcome in univariable analysis. The final list of variables included in the backward elimination regression modelling was: vegetation coverage, household crowding, household density, percentage of old buildings, percentage of degraded lodgings, percentage of apartments, percentage of lodgings with cement walls, unemployment (for 2008–2009 only), revenue (for 2012–2013 only) and age (average age of the population in each neighborhood).

Subsequently, a multivariable model containing the minimal number of explanatory variables was developed by backward elimination regression modelling. Variables that were the least strongly associated with the outcome (based on the largest p-value in the likelihood ratio test) were eliminated from the model in an iterative process until reaching a model containing only variables associated with the outcome (based on likelihood ratio test). For each epidemic, validation of the model was performed by comparing observed and predicted incidence rates and analyzing the residuals (see Supporting Information).

### Sensitivity analysis of the backward elimination modelling with an alternative variable categorization

To test whether categorization into quintiles introduced bias in the multivariable analysis, a sensitivity analysis was performed, during which backward elimination modelling was repeated with the same starting variables, but categorized in terciles instead of quintiles.

### Sensitivity analysis at a finer aggregation scale

For the 2008–2009 epidemic, dengue incidence rates and some socioeconomic data were available at a finer geographic aggregation unit corresponding to the population census (a block of houses with a median population of roughly 100 persons and an average surface area of 0.05 square-km, the “block of house scale”). Applying the modeling process used at the neighborhood scale to this smaller aggregation unit enabled us to perform a sensitivity analysis testing if risk factors identified for the 2008–2009 epidemic at the neighborhood scale are consistent with those found at a smaller scale. At that finer resolution, 25% of the dengue cases were lost due to misreported location. Data was not available to perform such a sensitivity analysis for the 2012–2013 epidemic.

## Results

### Comparing the 2008–2009 and 2012–2013 epidemics in Nouméa

In the city Nouméa, a total of 2,310 dengue cases were reported during the 2008–2009 epidemic (incidence rate: 23.7 cases per 1000 P-Y, 95% confidence interval (CI): 22.7–24.6). In Nouméa, the incidence rate was higher in 2012–2013 with 3,369 reported cases (34.5 cases/1000 P-Y, 95% CI: 33.4–35.7) (Table 2).

**Table 2:** Summary of the 2008–09 and 2012–13 epidemics in Nouméa.

	2008–09	2012–13
Circulating dengue serotypes	1 and 4	1
Dominant dengue serotype	4	1
Number of cases reported in New Caledonia	8,586	11,001
Number of cases reported in Nouméa	2,310	3,369
Incidence rate in Nouméa (95% CI)^(a)^	23.7 (22.7–24.6)	34.5 (33.4–35.7)
Peak time	March 2009	March 2013

^(a)^ cases per 1000 person-years, average over all neighborhoods

**Fig 1:**
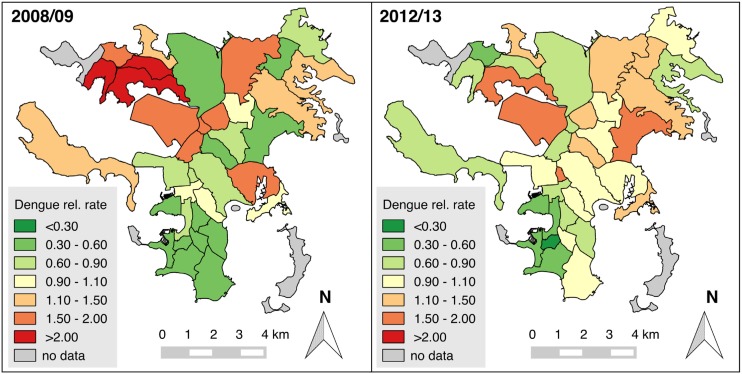
Relative dengue incidence rate in the neighborhoods of Nouméa during the 2008–2009 and 2012–2013 epidemics. The relative incidence rate was obtained by dividing the incidence rate in each neighborhood by the average incidence rate in the whole city. Shades of green indicate an incidence lower than average, shades of red indicate an incidence higher than average (see color scale).

Neighborhood-specific incidence rates ranged from 7.2 to 70.9 (cases/1000 P-Y) in 2008–2009 and from 6.4 to 60.6 in 2012–2013 (S1 Table). The relative incidence rates in each neighborhood (compared to the incidence throughout the entire city) seemed to follow a north-south gradient during 2008–2009 epidemic, but were distributed more homogeneously throughout the city during the 2012–2013 epidemic (Fig 1). This was confirmed by the presence of spatial autocorrelation in the distribution of the relative dengue incidence rates in 2008–2009 (global Moran’s I of 0.336, p<0.001, 95%CI: 0.183–0.489), but not in 2012–2013 (global Moran’s I of 0.036, p = 0.416, 95% CI: -0.053–0.125).

**Fig 2:**
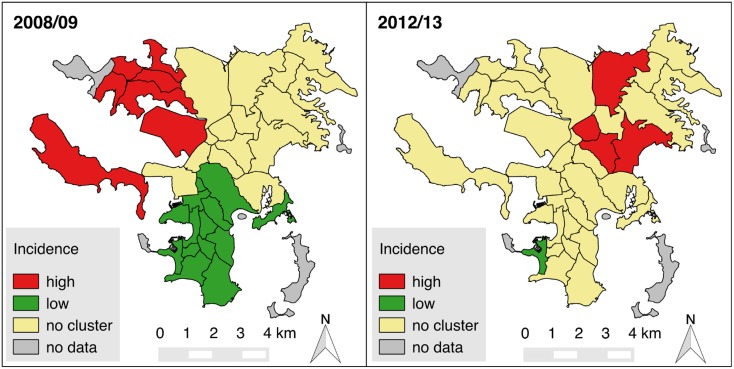
Clustering of high or low dengue incidence rate neighborhoods in the city of Nouméa. Clusters of neighborhoods of high (red) or low (green) incidence rate were detected using local Moran’s I statistics for the 2008–2009 or 2012–2013 epidemics.

Moreover, during the 2008–2009 epidemic, a cluster of high incidence neighborhoods in the northwest of the city (Numbo, Tindu, Ducos, Logicoop, Kaméré, Doniambo, Nouville) was detected (local Moran’s *I p*<0.05), while there was a large cluster of low incidence neighborhoods in the south (Artillerie, Quartier Latin, Vallée du Génie, Vallée des Colons, Faubourg Blanchot, Ouémo, Orphelinat, Trianon, N’Géa, Baie des Citrons, Receiving, Motorpool, Anse Vata, Val Plaisance, local Moran’s *I p*<0.05) (Fig 2). In 2012–2013, the spatial structure was less clear, with some high incidence neighborhoods clustering in the north of the city, and one low incidence neighborhood in the south.

### Factors associated with dengue incidence rate

**Fig 3:**
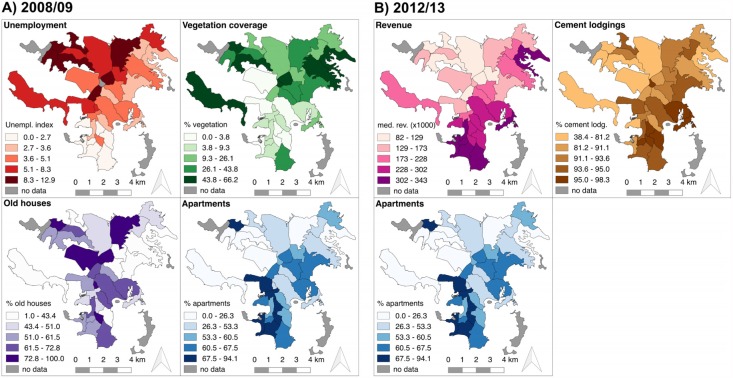
Distribution of variables associated with dengue incidence rate in 2008–2009 (A) and 2012–2013 (B). See text for details.

Univariable regression analysis identified 13 variables associated (*p*≤0.05) with dengue incidence rates during the 2008–2009 epidemic and 6 variables associated during the 2012–2013 epidemic (Table 3). The results of the multivariable analysis are presented in Table 4. In 2008–2009, dengue incidence rates were higher in neighborhoods with higher unemployment (IRR = 1.25, 95%CI: 1.14–1.36), higher vegetation coverage (IRR = 1.14, 95%CI: 1.04–1.24), higher percentage of old houses (IRR = 1.12, 95%CI: 1.03–1.21), and lower percentage of apartments (IRR = 0.91, 95%CI: 0.84–0.98). For 2012–2013, higher dengue incidence rates were associated with lower revenue (IRR = 0.88, 95%CI: 0.82–0.95), lower percentage of apartments (IRR = 0.91, 95% CI: 0.84–0.97) and higher percentage of cement lodgings (IRR = 1.13, 95% CI: 1.04–1.21). The distribution of these variables throughout the neighborhoods of Nouméa is represented in Fig 3, and the dengue incidence rates for all the quintiles of these variables are presented in S3 Fig.

**Table 3:** Results of the univariable regression analysis.

Variable	2008–09 epidemic		2012–13 epidemic	
	IRR (95% CI)^(a)^	*p*-value	IRR (95% CI)^(a)^	*p*-value
**Vegetation coverage**				
Vegetation coverage 2008	**1.30 (1.17–1.45)**	**<0.001**	n.a.	n.a.
Vegetation coverage 2013	n.a.^(b)^	n.a.	1.05 (0.97–1.14)	0.188
**House and people density**				
Household crowding	**1.22 (1.08–1.38)**	**0.002**	1.05 (0.96–1.14)	0.282
Household density	**0.82 (0.73–0.92)**	**0.001**	0.95 (0.87–1.03)	0.172
Population density	**0.88 (0.77–1.00)**	**0.049**	0.97 (0.89–1.05)	0.402
**Built environment**				
Old buildings	1.13 (1.00–1.28)	0.058	1.01 (0.93–1.09)	0.900
Degraded lodgings	0.97 (0.83–1.13)	0.706	1.06 (0.96–1.17)	0.232
Apartments	**0.83 (0.74–0.94)**	**0.004**	**0.92 (0.85–0.99)**	**0.033**
Cement lodgings	**0.84 (0.74–0.95)**	**0.005**	1.02 (0.94–1.11)	0.650
**Socioeconomic status**				
Unemployment	**1.41 (1.29–1.55)**	**<0.001**	**1.09 (1.00–1.18)**	**0.045**
Low education	**1.32 (1.18–1.47)**	**<0.001**	**1.09 (1.01–1.19)**	**0.024**
Revenue	**0.80 (0.71–0.90)**	**<0.001**	**0.90 (0.83–0.97)**	**0.004**
Difference in revenue	**0.77 (0.69–0.87)**	**<0.001**	**0.91 (0.84–0.98)**	**0.013**
Internet home access	**0.77 (0.69–0.85)**	**<0.001**	0.93 (0.86–1.02)	0.090
**Demographics**				
Born in Pacific	**1.33 (1.19–1.48)**	**<0.001**	**1.09 (1.01–1.18)**	**0.033**
Age	**0.83 (0.73–0.95)**	**0.004**	0.94 (0.87–1.02)	0.142

^(a)^ crude incidence rate ratio (IRR) and 95% confidence interval (incidence rate ratio is the relative increase in incidence when the explanatory variable increases from one quintile to the next)

^(b)^ not applicable

**Table 4:** Multivariable models for the 2008–09 and 2012–13 epidemics.

Variables	2008–09 epidemic^(a)^		20012–13 epidemic^(b)^	
	IRR (95%CI)^(c)^	*p*-value	IRR (95%CI)^(c)^	*p*-value
Unemployment	1.25 (1.14–1.36)	<0.001	n.a.	n.a.
Vegetation coverage	1.14 (1.04–1.24)	0.003	n.a.	n.a.
Old buildings	1.12 (1.03–1.21)	0.004	n.a.	n.a.
Apartments	0.91 (0.84–0.98)	0.016	0.91 (0.84–0.97)	0.005
Revenue	n.a.	n.a.	0.88 (0.82–0.95)	<0.001
Cement lodgings	n.a.	n.a.	1.13 (1.04–1.21)	0.001

^(a)^ adjusted for unemployment, vegetation coverage, old buildings and apartments

^(b)^ adjusted for revenue, apartments and cement lodgings

^(c)^ adjusted incidence rate ratio and 95% confidence interval

Details of model validation can be found in the Supporting Information and S4 Fig. The 2008–2009 multivariable model closely described the dengue incidence rates, including their spatial structure. The regression coefficient between the observed and predicted incidence rates was 0.73 (*p*<0.001, 95% CI: 0.57–0.89). For the 2012–2013 epidemic, our model reliably associated some variables with dengue incidence, but failed to fully describe the variation of incidence rate across the different neighborhoods. The regression coefficient between the observed and predicted incidence rates was only 0.33 (*p*<0.001, 95% CI: 0.16–0.51). Moreover, for 2012–2013, residuals were positively correlated with incidence rates, suggesting that some important explicative variable may be missing.

### Sensitivity analysis

The model resulting from backward elimination modelling using variables categorized in terciles is presented in S4 Table. For 2008–2009, the sensitivity analysis confirmed the association between vegetation coverage and unemployment, but percentage of old houses and percentage of apartments were not present in the final model. For 2012–2013, median revenue, percentage of apartments and percentage of cement lodgings were all associated with dengue incidence rates, similarly to the model using variables in quintiles. For both epidemics, the original model using variables categorized in quintiles had a better fit (lower AIC) compared to the model using variables categorized in terciles.

The result of the multivariable model obtained for 2008–09 using data available at a smaller aggregation unit (“block of house scale”) is presented in S5 Table. Percentage of residents born in the Pacific Island (highly correlated to the unemployment index), vegetation coverage and percentage of apartments remained significantly associated at this finer scale. However, the association with vegetation coverage was reversed at this smaller scale, with an IRR lesser than one (S5 Table).

## Discussion

We performed an ecological study to investigate the association between dengue incidence during two major dengue epidemics (2008–2009 and 2012–2013) and various socioeconomic and environmental factors in Nouméa. The 2008–2009 epidemic featured a more pronounced geographic variation in dengue incidence across the city, with high incidence neighborhoods in the north, and low incidence neighborhoods in the south. In contrast, the 2012–2013 epidemic was more homogenously distributed throughout the city. We found that incidence rates were associated with socioeconomic and environmental factors during both epidemics.

Our results are consistent with previous studies from the Americas that demonstrated in urban settings an association between dengue incidence or mosquito density and revenue, unemployment, vegetation coverage, housing quality and/or percentage of apartments [33, 37–39, 43, 46–49, 54]. Our results expand those findings by showing that similar socioeconomic factors influence dengue distribution in a very different geographical context (an island in the Pacific) and in a much smaller city (Nouméa). This suggests that the influence of socioeconomic determinants on dengue transmission may be a general phenomenon. However, the reasons why low socioeconomic status is linked to dengue incidence may be location-specific, because the cause and consequences of having a low socioeconomic status may differ widely between locations. Therefore, further local studies are necessary to understand the connection between low socioeconomic status and dengue incidence rates in different settings.

Neighborhoods in which people had a lower socioeconomic status (high unemployment, low revenue or high percentage of people born in the Pacific, which are all related) were associated with higher dengue incidence rates during both epidemics, and in all models developed (main model, model with different variable categorization, and model with finer aggregation unit). This suggests that the socioeconomic status of the population is a very robust correlate of dengue incidence. In Nouméa, possible explanations include the fact that unemployment may induce lifestyle behaviors that increase the risk of mosquito contact, for example spending more time in and around the house during the day, when mosquitoes are also known to bite [39]. Furthermore, being born in the Pacific may result in different habits concerning personal protection against mosquitoes. Recently, a country-wide ecological spatial study in New Caledonia showed a similar association between unemployment and higher dengue incidence rates [71]. The robust association between socioeconomic status and dengue incidence underscores the importance of human behavior in modulating the transmission risk, and highlights the need to perform social studies to better understand high-risk behaviors.

Variables related to the built and natural environment (proportion of apartments in all lodgings, proportion of old houses, proportion of cement lodgings, vegetation coverage) were also associated with dengue incidence, but the factors associated were not always the same during both epidemics and across all models. This suggests that environmental factors may not correlate with dengue incidence as consistently as socioeconomic status. Nevertheless, a higher proportion of apartments in all lodgings was associated with lower dengue incidence during both epidemics, and in all models but one. One can speculate that reduced garden space in apartment complexes results in fewer breeding sites and mosquitoes [54] and induce a more indoors lifestyle, where mosquito contact is less likely [55]. In 2008–2009, a higher percentage of old houses was positively associated with dengue incidence rate. Perhaps older houses lack equipment such as air-tight windows, and air conditioning, which all reduce the number of mosquitoes found inside the house, as suggested previously [37].

While vegetation coverage was positively associated with dengue in 2008–09 using the neighborhood as aggregation unit, it was associated with lower incidence at the finer “block of house” aggregation scale. This is an example of the “modifiable area unit problem” [72–75], which suggests that analyzing the same data using another size and/or shape of the aggregation units can generate different results. Perhaps due to the limited flight range of *Aedes aegypti* mosquitoes (usually remaining within a 400m radius circle during their lifetime [76]), association with vegetation coverages is different at this smaller aggregation unit. Alternatively, it could be due to the use of different databases (25% missing cases at the “block of house scale”).

In 2012–2013, dengue incidence rates were positively associated with the percentage of cement lodging. The association between this variable and dengue incidence rates is characterized by a threshold effect, with a sharp increase in incidence rates from the first to the second quintile, and a smooth decrease afterwards (S3 Fig). Fitting one IRR for all quintiles captures the increase in incidence, which is likely to be caused by reporting bias. Indeed, people living in non-cement lodging (lowest quintile) may be less prone to visit health services, resulting in an artificially low reported dengue incidence rate for that quintile. It also appears that our model for the 2012–2013 epidemic may be missing a variable, causing residual confounding.

The spatial pattern of dengue incidence differed markedly between the two epidemics: with a north-south gradient in incidence rates for 2008–2009 and widely homogenous incidence rates for 2012–2013, perhaps explaining why associations with socioeconomic factors differed between the two epidemics. The 2008–2009 epidemic was unusual because it was characterized by the prolonged co-circulation of two serotypes: first DENV1, which had been circulating in the Pacific Islands for years, replaced later by DENV4, which had not been reported in the Pacific for over 20 years but caused most of the cases [19]. The 2012–2013 epidemic, in contrast, was dominated by DENV1, which had already caused a major epidemic in 2002–03 [19]. The difference in circulating serotypes may contribute to the observed differences in spatial structure between both epidemics, as it has been previously suggested that dengue clustering patterns within a city are serotype-dependent [31]. In addition, DENV1 had been circulating regularly in the Pacific region and in New Caledonia while DENV4 re-emerged in 2008–2009 after decades of absence. Therefore, the pre-epidemic immunological status of the population was certainly different for DENV1 and for DENV4 (population immunity to DENV4 was probably lower than for DENV1), which is likely to have influenced the spatial distribution of dengue cases, and possibly blurred association with socioeconomic factors during the 2012–13 epidemic.

Another limitation is that surveillance based on dengue cases visiting healthcare facilities may underestimate the true number of cases in the population, which could bias the analysis if this underestimation was heterogeneous across the different neighborhoods. Health care is free in New Caledonia, so financial barriers to using health care services should be low, but some people (e.g. long-time residents familiar with dengue symptoms) may judge it unnecessary to seek medical attention. Also, asymptomatic dengue infections, which can influence transmission patterns [77], were not captured as the surveillance system was based on reporting of clinical cases. The immune status of the population is another important determinant of dengue transmission, as dengue immunity confers protection against subsequent infection with a homologous serotype, but can exacerbate disease severity upon re-infection with another serotype [78, 79]. Ideally, population immunity should be included in a model of dengue transmission, but this data was not available for our study.

As vaccine development efforts are still ongoing, education and vector control remain important components of dengue prevention. In New Caledonia, prevention measures include (i) regular visit to individual houses to inform about dengue, (ii) monitoring and elimination of mosquito breeding sites (iii) education about dengue as part of regular school programs and (iv) residual spraying of houses 100 meters around cases [61]. Our results suggest that during or before dengue epidemics, prevention efforts should be directed in priority towards neighborhoods of lower socioeconomic status, and/or areas with large vegetation coverage around individual houses.

## Supporting information

S1 Text Estimation of vegetation coverage and model validation. (DOCX)

S1 Fig Study site. See text for details.
(EPS)

S2 Fig Estimation of the area covered by vegetation during the two dengue epidemics. See supplementary information for details.
(TIFF)

S3 Fig Dengue incidence rates for each quintile of the variables associated with the 2008–09 (left) and 2012–13 (right) epidemics. Each boxplot represents the first, second and third quartiles of incidence rates across neighborhoods in the quintile of the explanatory variable, whiskers represent 1.5 of the inter-quartile range, empty dots are outliers (neighborhoods with incidence rates outside the inter-quartile range), filled triangle is the mean incidence rate across all neighborhoods in the quintile.
(EPS)

S4 Fig Model validation for 2008–09 and 2012–13 epidemics. (A) Association between observed and predicted incidence rates with regression line (dotted line) and regression coefficient (with 95% CI); the solid line has a slope of 1. (B) Distribution of residuals obtained by subtracting predicted from observed incidence rate and plotted as a histogram. (C) Association between residuals and incidence rate, with regression line (dotted line) and regression coefficient (with 95% CI).
(EPS)

S1 Table Geographical characteristics and incidence rates for all neighborhoods. (DOCX)

S2 Table Variables used in regression analysis and their quintiles. (DOCX)

S3 Table Pearson correlation between dependent variables. The lower triangle of the tables shows correlation coefficients whereas the upper triangle shows p-values.
(DOCX)

S4 Table Multivariable models for the 2008–09 and 2012–13 epidemics, with variables categorized in terciles. (DOCX)

S5 Table Multivariable model for the 2008–09 epidemic, with data aggregated at the block of houses scale. (DOCX)
