# Supplementary material for: Socioeconomic and environmental determinants of dengue transmission in an urban setting: An ecological study in Nouméa, New Caledonia
Source: PLoS Negl Trop Dis. 2017 Apr 3;11(4):e0005471. doi: 10.1371/journal.pntd.0005471 (PMC5395238; doi:10.1371/journal.pntd.0005471)
Supplement: S1 Text — (DOCX) [file pntd.0005471.s001.docx]

**Determinants of urban dengue transmission**

Zellweger et al.

**Supporting Information**

**Estimation of vegetation coverage during the two dengue epidemics**

High resolution remote sensing data were used to estimate surface area covered by vegetation in Nouméa during the dengue outbreaks. Cloud-free Landsat 7 ETM+ images covering Nouméa and surrounding areas were obtained from the U.S. Geological Survey (USGS) website [1] for October 2008 (2008/10/06) and February 2013 (2013/02/06) (S2 Fig). Unfortunately, the Landsat 7 ETM+ sensor had a failure on May 31^st^ 2003 which affected its scan-line corrector (SLC) system, a mechanism designed to compensate for the satellite’s forward motion during image acquisition[2]. This failure ultimately causes roughly 22% of the pixels to be un-scanned in all ETM+ images from this date onwards. Moreover, gaps are produced in a zig-zag pattern and the width of these wedge-shaped data gaps increases the further east or west from the nadir path, resulting in large missing areas toward the edges of the scene, which leaves a major problem for the applications of ETM+ images (S2 Fig A, B). Several methods have been designed to fill this gaps. We opted to apply a deterministic interpolation approach developed by the USGS, referred to as local linear histogram-matching method [3]. For this, additional suitable images of the same scene; cloud free, similar atmospheric conditions and nearest in time to the images to be corrected (Oct-2011 & Mar-2012), were also downloaded from USGS. After filling the gaps on the Landsat 7 SLC-off images, we proceeded to combine the multispectral bands (provided at 30m resolution) and the 15-m panchromatic band through a pan-sharpening process to increase their spatial resolution. Atmospheric correction was then conducted using the DOS model-based algorithm and assuming 1% minimum reflectance [4]. Colour infrared (CIR) composite images were created to highlight vegetated areas, combining near-infrared band (band 4), red band (band 3) and green band (band 2) (S2 Fig C, D). Subsequently, vegetated areas were discriminated from water and built-up areas by implementing a supervised classification approach based on the maximum likelihood algorithm (S2 Fig E, F) [5]. Training areas were identifying for each type of coverage over the CIR images and their reliability to discriminate each type of land cover was roughly cross-checked using finer spatial resolution images (i.e. IKONOS and Quickbird) available from Google Earth.

Percentage of surface area covered by vegetation was finally estimated for each Nouméa neighborhood by overlaying the classified images and a vector layer of neighborhoods.

**Validation of the multivariable models**

Model performance was assessed by calculating the correlation coefficient between observed incidence rates and incidence rates predicted by the multivariable models. The correlation coefficient was 0.73 (*p*<0.001, 95% CI: 0.57-0.89) for 2008-09, suggesting a good correlation between observed and predicted values. In 2012-13, the correlation coefficient was 0.33 (*p*<0.001, 95% CI: 0.16-0.51) (S4 Fig A).

Validation of model assumptions was performed by checking independence, normality and homoscedasticity of residuals. A histogram of the residuals of each multivariable models is presented in S4 Fig B. For one neighborhood (Montagne Coupée) the unemployment index was not available. Therefore, for this neighborhood, no residual was calculated in 2008/09 because incidence rate could not be predicted. According to the Shapiro test, the residuals followed a normal distribution for both models (2008-09: *p*=0.598, 2012/13: *p*=0.292). Levene’s test performed on residuals (divided in three groups) did not show any evidence of heteroscedasticity (2008-09: *p*=0.476, 2012-13: *p*=0.877). The slope of the regression line between the residuals in the incidence rate was 0.27 (p=0.001, 95% CI: 0.11-0.43) in 2008/09 and 0.67 (*p*<0.001, 95% CI: 0.49-0.84) in 2012-13, suggesting a weak linear relationship between residuals and observed incidence in 2008-09, and a stronger one in 2012-2013 (S4 Fig C).

Finally, the spatial autocorrelation of the residuals was assessed by calculating the global Moran’s *I* statistic. For the 2008-09 multivariable model, the global Moran’s *I* statistics of the residuals was 0.015 (*p*=0.764, 95% CI: -0.084-0.113). The absence of spatial structure in the residuals suggests that our multivariable model adequately accounted for the spatial structure observed in dengue incidence rate in 2008/09, and that the auto-correlation observed in incidence rates was induced by autocorrelation in the independent variables. The residuals of the 2012-13 model also lacked spatial structure (global Moran’s *I* statistic = 0.002, *p*=0.935, 95% CI: -0.056-0.060), as did the incidence rates during that epidemic.

**Supplementary References**

1. USGS, <http://earthexplorer.usgs.gov/> (accessed May 2015).

2. Arvidson T, Goward S, Gasch J, Williams D. Landsat-7 Long-Term Acquisition Plan. Photogrammetric Engineering & Remote Sensing. 2006;72(10):1137-46. doi: 10.14358/PERS.72.10.1137.

3. USGS. Phase 2 gap-fill algorithm: SLC-off gap-filled products gap-fill algorithm methodology. 2004.

4. Zhang Z, He G, Wang X. A practical DOS model-based atmospheric correction algorithm. International Journal of Remote Sensing. 2010;31(11):2837-52. doi: 10.1080/01431160903124682.

5. Keuchel J, Naumann S, Heiler M, Siegmund A. Automatic land cover analysis for Tenerife by supervised classification using remotely sensed data. Remote Sensing of Environment. 2003;86(4):530-41. doi: <http://dx.doi.org/10.1016/S0034-4257(03)00130-5>.
