# Supplementary figures and images for: Socioeconomic and environmental determinants of dengue transmission in an urban setting: An ecological study in Nouméa, New Caledonia
Source: PLoS Negl Trop Dis. 2017 Apr 3;11(4):e0005471. doi: 10.1371/journal.pntd.0005471 (PMC5395238; doi:10.1371/journal.pntd.0005471)

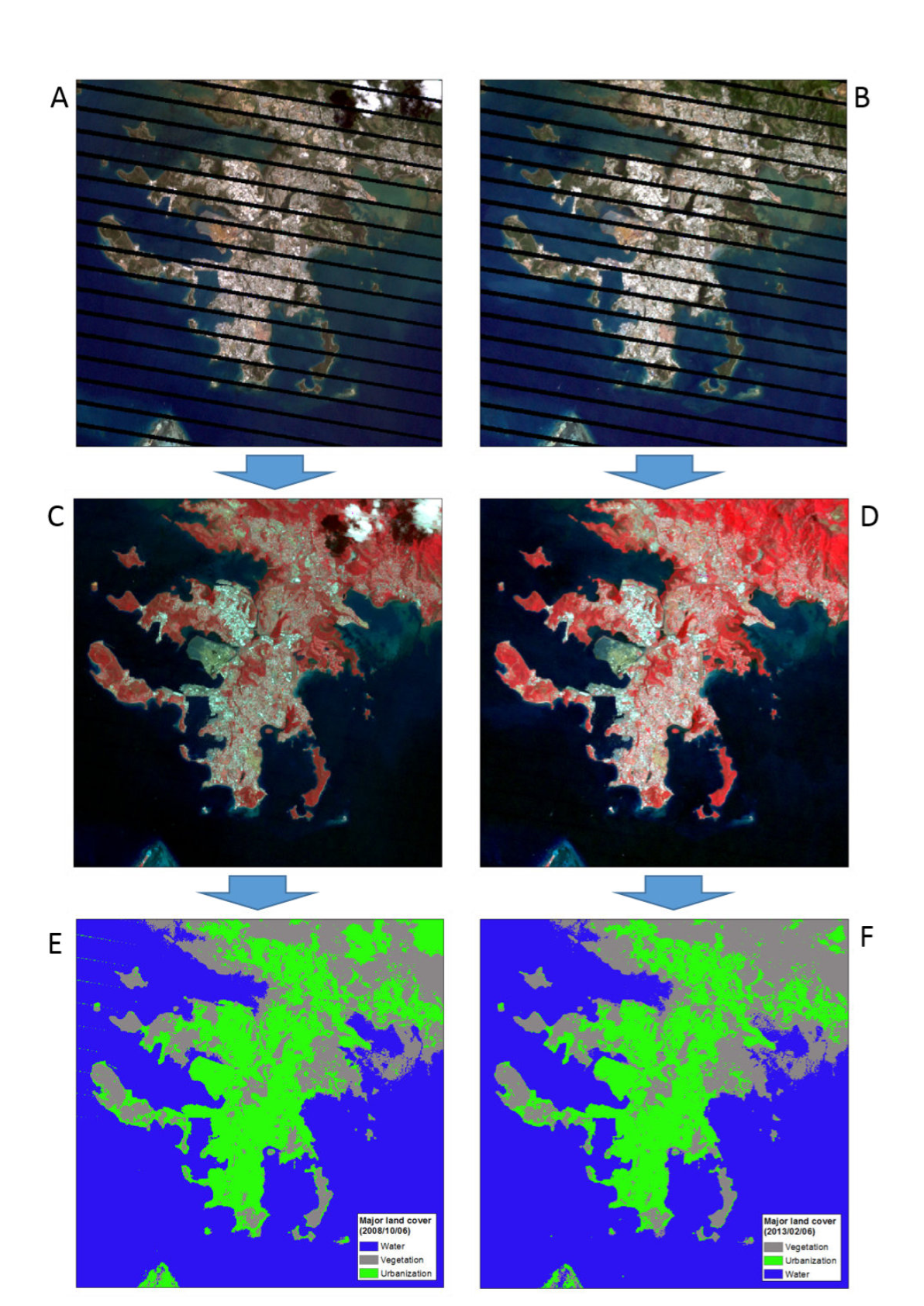

Supplement: S2 Fig — See supplementary information for details. (TIFF) [file pntd.0005471.s003.tiff]
