# Supplementary material for: Socioeconomic and environmental determinants of dengue transmission in an urban setting: An ecological study in Nouméa, New Caledonia
Source: PLoS Negl Trop Dis. 2017 Apr 3;11(4):e0005471. doi: 10.1371/journal.pntd.0005471 (PMC5395238; doi:10.1371/journal.pntd.0005471)
Supplement: S1 Table — (DOCX) [file pntd.0005471.s006.docx]

**S1 Table 1. Geographical characteristics and incidence rates for all neighborhoods**

|  | **Population** | **Surface** | **Population** | **Dengue Incidence Rate**^(b)^ | |
| --- | --- | --- | --- | --- | --- |
| **Neighborhood** | **in 2009** | **Area (km^2^)** | **Density**^(a)^ | **2008-2009** | **2012-2013** |
| Aérodrome | 4,687 | 2.30 | 2,039 | 9.0 | 53.3 |
| Anse Vata | 3,187 | 1.02 | 3,112 | 10.7 | 19.5 |
| Artillerie | 1,846 | 0.54 | 3,407 | 7.6 | 12.5 |
| Baie des citrons | 2,235 | 0.51 | 4,381 | 7.2 | 14.8 |
| Centre ville | 2,174 | 1.41 | 1,539 | 17.5 | 35.4 |
| Doniambo | 279 | 2.78 | 100 | 35.8 | 57.3 |
| Ducos | 2,231 | 1.75 | 1,278 | 58.3 | 52.9 |
| Ducos zone industrielle | 1,428 | 4.14 | 345 | 10.5 | 26.6 |
| Faubourg Blanchot | 2,816 | 0.69 | 4,078 | 21.3 | 34.8 |
| Haut Magenta | 2,310 | 0.77 | 3,015 | 13.9 | 41.1 |
| Kaméré | 3,138 | 0.80 | 3,906 | 27.7 | 39.8 |
| Koumourou^(c)^ | 0 | 1.17 | 0 | n.a. | n.a. |
| Logicoop | 1,365 | 0.83 | 1,636 | 52.7 | 30.8 |
| Magenta | 7,566 | 1.71 | 4,416 | 36.1 | 35.0 |
| Montagne coupée | 252 | 0.17 | 1,467 | 43.7 | 23.8 |
| Montravel | 2,403 | 0.67 | 3,565 | 40.8 | 47.0 |
| Motor Pool | 2,021 | 0.36 | 5,676 | 11.9 | 32.2 |
| N'Géa | 2,848 | 0.88 | 3,237 | 9.5 | 30.2 |
| Normandie | 4,550 | 1.76 | 2,590 | 20.7 | 32.5 |
| Nouville | 1,952 | 4.51 | 433 | 32.3 | 28.7 |
| Numbo | 254 | 0.86 | 296 | 70.9 | 23.6 |
| Orphelinat | 1,236 | 0.48 | 2,571 | 14.6 | 13.8 |
| Ouemo | 1,501 | 0.63 | 2,386 | 22.0 | 47.3 |
| Portes de fer | 4,242 | 1.12 | 3,788 | 18.2 | 36.8 |
| Quartier Latin | 1,525 | 0.46 | 3,345 | 22.3 | 28.2 |
| Quatrième kilomètre | 2,642 | 0.98 | 2,690 | 24.2 | 32.9 |
| Receiving | 314 | 0.25 | 1,234 | 12.7 | 6.4 |
| Rivière salée | 8,772 | 3.03 | 2,893 | 36.0 | 38.4 |
| Septième kilomètre | 3,608 | 1.01 | 3,586 | 10.3 | 39.6 |
| Sixième kilomètre | 2,821 | 2.81 | 1,005 | 26.2 | 40.4 |
| Tina | 1,769 | 1.96 | 902 | 33.4 | 25.4 |
| Tindu | 1,994 | 0.63 | 3,189 | 46.1 | 16.0 |
| Trianon | 2,663 | 0.44 | 5,989 | 8.3 | 25.9 |
| Val Plaisance | 3,346 | 1.75 | 1,915 | 12.0 | 37.4 |
| Vallée des colons | 9,372 | 2.12 | 4,431 | 20.6 | 35.2 |
| Vallée du génie | 264 | 0.13 | 2,019 | 18.9 | 60.6 |
| Vallée du tir | 1,968 | 0.64 | 3,059 | 42.7 | 30.5 |

(a) people/km^2^

(b) dengue cases per 1000 person-years

(c) Koumourou has no inhabitants, and therefore was excluded of the analysis
