# Supplementary material for: Socioeconomic and environmental determinants of dengue transmission in an urban setting: An ecological study in Nouméa, New Caledonia
Source: PLoS Negl Trop Dis. 2017 Apr 3;11(4):e0005471. doi: 10.1371/journal.pntd.0005471 (PMC5395238; doi:10.1371/journal.pntd.0005471)
Supplement: S2 Table — (DOCX) [file pntd.0005471.s007.docx]

**S2 Table.** **Variables used in regression analysis and their quintiles**

|  | | **Range of quintiles** | | | | |
| --- | --- | --- | --- | --- | --- | --- |
| **Variable** | | **Quintile 1** | **Quintile 2** | **Quintile 3** | **Quintile 4** | **Quintile 5** |
| **Vegetation coverage** | |  |  |  |  |  |
|  | Vegetation coverage 2008 (% area) | 0.0-3.5 | 3.8-8.2 | 9.3-21.4 | 26.1-41.9 | 43.8-66.2 |
|  | Vegetation coverage 2013 (% area) | 0.0-3.8 | 4.0-9.1 | 9.4-26.9 | 27.5-41.5 | 44.3-64.6 |
| **House and people density** | |  |  |  |  |  |
|  | Household crowding (people/room) | 0.65-0.70 | 0.70-0.79 | 0.80-0.91 | 0.93-1.11 | 1.12-2.4 |
|  | Household density (household/km^2^) | 23-319 | 489-678 | 703-995 | 1017-1361 | 1381-2599 |
|  | Population density (people/km^2^) | 100-1234 | 1278-2039 | 2386-3059 | 3112-3586 | 3788-5989 |
| **Built environment** | |  |  |  |  |  |
|  | Old buildings (% of all buildings) | 1.0-33.2 | 43.4-50.7 | 51.0-60.0 | 61.5-72.7 | 72.8-100 |
|  | Degraded lodgings (% of all lodgings) | 0-1.2 | 1.6-3.8 | 4.1-7.3 | 7.5-32.3 | n.a. ^(b)^ |
|  | Apartments (% of all lodgings) | 0-25.6 | 26.3-49.9 | 53.3-60.2 | 60.5-66.5 | 67.5-94.1 |
|  | Cement lodgings (% of all lodgings) | 38.4-79.5 | 81.2-90.5 | 91.1-93.5 | 93.6-95.0 | 95.0-98.3 |
| **Socio-economic status** | |  |  |  |  |  |
|  | Unemployment (% of active-age pop.) | 0-2.6 | 2.7-3.5 | 3.6-5 | 5.2-8.2 | 8.9-12.9 |
|  | Low education (% of population) | 29.8-35.7 | 36.6-50.6 | 51.6-60.4 | 64.2-77.8 | 77.9-89.6 |
|  | Revenue (in thousands FCFP^(a)^) | 82-125 | 129-171 | 173-224 | 228-287 | 302-343 |
|  | Difference in revenue (in thousands FCFP^(a)^) | 189-306 | 316-389 | 397-488 | 502-624 | 627-814 |
|  | Internet home access (% of all households) | 4.4-23.2 | 26.6-40.4 | 41.2-55.3 | 55.4-68.1 | 69.9-81 |
| **Demographics** | |  |  |  |  |  |
|  | Born in Pacific (% of population) | 22.0-42.9 | 43.0-61.0 | 61.3-72.8 | 75.8-86.0 | 89.3-98.9 |
|  | Age (average age of neighborhood) | 25.3-29.9 | 30.2-32.6 | 32.7-34.0 | 34.0-37.1 | 37.6-43.2 |

^(a)^ in Franc des Colonies Françaises du Pacifique (FCFP), 1000 FCFP ≈ 8.5 € at the time of writing

^(b)^ not available: no upper quintile, because 12 neighborhoods had a value of 0 for that variable and were grouped in the lowest quintile
