# Supplementary material for: Socioeconomic and environmental determinants of dengue transmission in an urban setting: An ecological study in Nouméa, New Caledonia
Source: PLoS Negl Trop Dis. 2017 Apr 3;11(4):e0005471. doi: 10.1371/journal.pntd.0005471 (PMC5395238; doi:10.1371/journal.pntd.0005471)
Supplement: S3 Table — The lower triangle of the tables shows correlation coefficients whereas the upper triangle shows p-values. (DOCX) [file pntd.0005471.s008.docx]

**S3 Table. Pearson correlation between dependent variables.** The lower triangle of the tables shows correlation coefficients whereas the upper triangle shows p-values.

3A. Variables linked to socio-economic status:

|  | **Unemployment** | **Low education** | **Revenue** | **Difference in revenue** | **Internet home access** | **Born in the Pacific** |
| --- | --- | --- | --- | --- | --- | --- |
| **Unemployment** | **1.00** | <0.05 | <0.05 | <0.05 | <0.05 | <0.05 |
| **Low education** | **0.83** | **1.00** | <0.05 | <0.05 | <0.05 | <0.05 |
| **Revenue** | **-0.78** | **-0.92** | **1.00** | <0.05 | <0.05 | <0.05 |
| **Difference in revenue** | **-0.76** | **-0.92** | **0.94** | **1.00** | <0.05 | <0.05 |
| **Internet home access** | **-0.84** | **-0.97** | **0.90** | **0.87** | **1.00** | <0.05 |
| **Born in the Pacific** | **0.75** | **0.95** | **-0.84** | **-0.88** | **-0.90** | **1.00** |

3B. Variables linked to population and household density:

|  | **Household density** | **Population density** | **Household crowding** |
| --- | --- | --- | --- |
| **Household density** | **1.00** | <0.05 | <0.05 |
| **Population density** | **0.93** | **1.00** | 0.05 |
| **Household crowding** | -0.46 | -0.33 | **1.00** |

3C. Variables linked to the built environment:

|  | **Old buildings** | **Degraded lodgings** | **Apartments** | **Cement lodgings** |
| --- | --- | --- | --- | --- |
| **Old buildings** | **1.00** | 0.05 | 0.59 | 0.54 |
| **Degraded lodgings** | 0.33 | **1.00** | 0.24 | 0.37 |
| **Apartments** | 0.09 | 0.20 | **1.00** | <0.05 |
| **Cement lodgings** | 0.11 | 0.15 | 0.55 | **1.00** |
