# Supplementary material for: Socioeconomic and environmental determinants of dengue transmission in an urban setting: An ecological study in Nouméa, New Caledonia
Source: PLoS Negl Trop Dis. 2017 Apr 3;11(4):e0005471. doi: 10.1371/journal.pntd.0005471 (PMC5395238; doi:10.1371/journal.pntd.0005471)
Supplement: S4 Table — (DOCX) [file pntd.0005471.s009.docx]

**S4 Table. Multivariable models for the 2008-09 and 2012-13 epidemics,**

**with variables categorized in terciles**

|  | **2008-09 epidemic^(a)^** | |  | **20012-13 epidemic^(b)^** | |
| --- | --- | --- | --- | --- | --- |
| **Variables** | **IRR (95%CI)^(c)^** | ***p*-value** |  | **IRR (95%CI)** | ***p*-value** |
| Unemployment | 1.55 (1.31-1.84) | <0.001 |  | n.a. | n.a. |
| Vegetation coverage | 1.30 (1.10-1.54) | 0.002 |  | n.a. | n.a. |
| Apartments | n.a. | n.a. |  | 0.83 (0.72-0.96) | 0.009 |
| Revenue | n.a. | n.a. |  | 0.83 (0.72-0.97) | 0.012 |
| Cement lodgings | n.a. | n.a. |  | 1.19 (1.03-1.38) | 0.015 |
