# Supplementary material for: Socioeconomic and environmental determinants of dengue transmission in an urban setting: An ecological study in Nouméa, New Caledonia
Source: PLoS Negl Trop Dis. 2017 Apr 3;11(4):e0005471. doi: 10.1371/journal.pntd.0005471 (PMC5395238; doi:10.1371/journal.pntd.0005471)
Supplement: S5 Table — (DOCX) [file pntd.0005471.s010.docx]

**S5 Table.** **Multivariable model for the 2008-09 epidemic,**

**with data aggregated at the block of houses scale**

|  | **2008-09 epidemic** | | |
| --- | --- | --- | --- |
| **Variables** | **IRR (95%CI)^(a)^** | ***p*-value** |  |
| % born in New-Caledonia | 1.19 (1.14-1.27) | <0.001 |  |
| Vegetation coverage | 0.92 (0.88-0.97) | 0.0014 |  |
| Apartments | 0.88 (0.85-0.92) | <0.001 |  |

^(a)^ incidence rate ratio and 95% confidence interval
